# Medical Thoracoscopy in Malignant Pleural Effusion

**DOI:** 10.7759/cureus.85707

**Published:** 2025-06-10

**Authors:** Amit K Rath, Sudarsan Pothal, Pallavi Bharadwaj

**Affiliations:** 1 Pulmonary Critical Care and Sleep Medicine, Srirama Chandra Bhanja (SCB) Medical College and Hospital, Cuttack, IND; 2 Department of Respiratory Medicine, Shri Jagannath Medical College and Hospital, Puri, IND; 3 Department of Physiology, Institute of Medical Sciences (IMS) and SUM Hospital II, Bhubaneswar, IND

**Keywords:** malignant pleural effusion, mass, medical thoracoscopy, nodule, plaque

## Abstract

Introduction: Malignant pleural effusion (MPE) is a common complication of advanced malignancies, often resulting in significant morbidity and impaired quality of life. In most cases, pleural fluid cytology remains negative. In the era of targeted cancer therapy, pathologists expect larger tissue samples. Therefore, medical thoracoscopy (MT) has gained popularity for adequate tissue sampling under supervision. During MT, various morphological pleural lesions were identified, yet their histopathological associations remain unknown. This study aimed to assess the diagnostic yield of medical thoracoscopy and the complications associated with the procedure. We also observed various morphological lesions in MPE and their histopathological associations.

Methods: This was a single-center, hospital-based, prospective cross-sectional study conducted in the Department of Pulmonary Medicine, Veer Surendra Sai Institute of Medical Sciences and Research (VIMSAR), Burla, India. Patients with undiagnosed pleural effusion underwent MT. During the procedure, the lesions were morphologically characterized, and samples were collected for histopathological examination (HPE).

Results: A total of 87 patients underwent medical thoracoscopy using a rigid thoracoscope (Optymed®, Delhi, India), out of which 72 patients had malignant effusion and seven patients had tuberculous effusion. Eight patients had nonspecific inflammation. The diagnostic yield of medical thoracoscopy in this study was at least 90.8% (95% CI: 84.8%-96.8%). Seventy-two patients were evaluated for histopathologic and morphologic association. The most common procedure-related complication was pain at the incision site (87, 100%, median Visual Analogue Scale (VAS): 5), followed by desaturation. The mean fall in saturation was 5.13% (±33.78%). We did not observe any serious adverse effects related to rigid MT. The mean age of the patients was 54.17 (±15.17) years. Among MPE, pleural-based nodules (46, 63.9%), followed by adhesions (27, 37.5%), were the most common findings during MT. Other noted lesions included masses (26, 36.12%), plaques (22, 30.56%), and strands (19, 26.39%). Nodules were significantly associated with metastatic adenocarcinoma (p<0.01). Adhesions and plaques were commonly linked with pleural abnormalities in malignant mesothelioma (p<0.01). Masses were most frequently associated with lymphomas (p<0.01) and undifferentiated carcinoma (p<0.01). Small cell carcinoma comprised only five patients among MPE and was associated with strands (p<0.01).

Conclusion: Medical thoracoscopy is a valuable tool in evaluating and managing malignant pleural effusion. Its high diagnostic accuracy, therapeutic capabilities, and favorable safety profile make it the procedure of choice in appropriate clinical settings. Specific morphological patterns may be observed in different pleural malignancies.

## Introduction

Pleural effusions have been encountered by pulmonologists in day-to-day clinical practice. Malignant pleural effusion (MPE) is a frequent and debilitating manifestation of advanced malignancies. Pleural malignancy is the second leading cause of exudative pleural effusion after parapneumonic effusion [1]. The most common malignancies associated with MPE are carcinoma of the lungs, carcinoma of the breast, lymphoma, and metastatic adenocarcinoma of the ovaries [2]. Despite high clinical suspicion, the yield of pleural fluid cytology is low [3,4]. Medical thoracoscopy (MT) is useful for detecting pleural neoplasia with negative pleural fluid cytology reports [5]. The main advantage of medical thoracoscopy is obtaining biopsy samples under direct visualization of the pleura. Various pleural morphological lesions are mentioned in different studies, such as plaques, mass lesions, nodules, adhesions, and fibrinous strands [6-8]. However, the histopathological associations of these lesions remain unknown. This study aimed to measure the diagnostic yield of medical thoracoscopy and its associated complications. We also emphasized the importance of lesion characteristics and their association with different malignancies.

## Materials and methods

The study was conducted from January 2016 to December 2018 in the Department of Pulmonary Medicine, Veer Surendra Sai Institute of Medical Sciences and Research (VIMSAR), Burla, Odisha. It was a hospital-based, single-center, prospective, cross-sectional study. We followed the ethics guidelines for biomedical research on human subjects by the Central Ethics Committee on Human Research (CECHR), ICMR-2000, and those contained in the Declaration of Helsinki. The Institutional Ethics Committee approved the study trial (approval 2015/P-I-RP/131). Written consent to participate and future publication of the retrieved data was obtained from all study participants.

Study population

**Figure 1:**
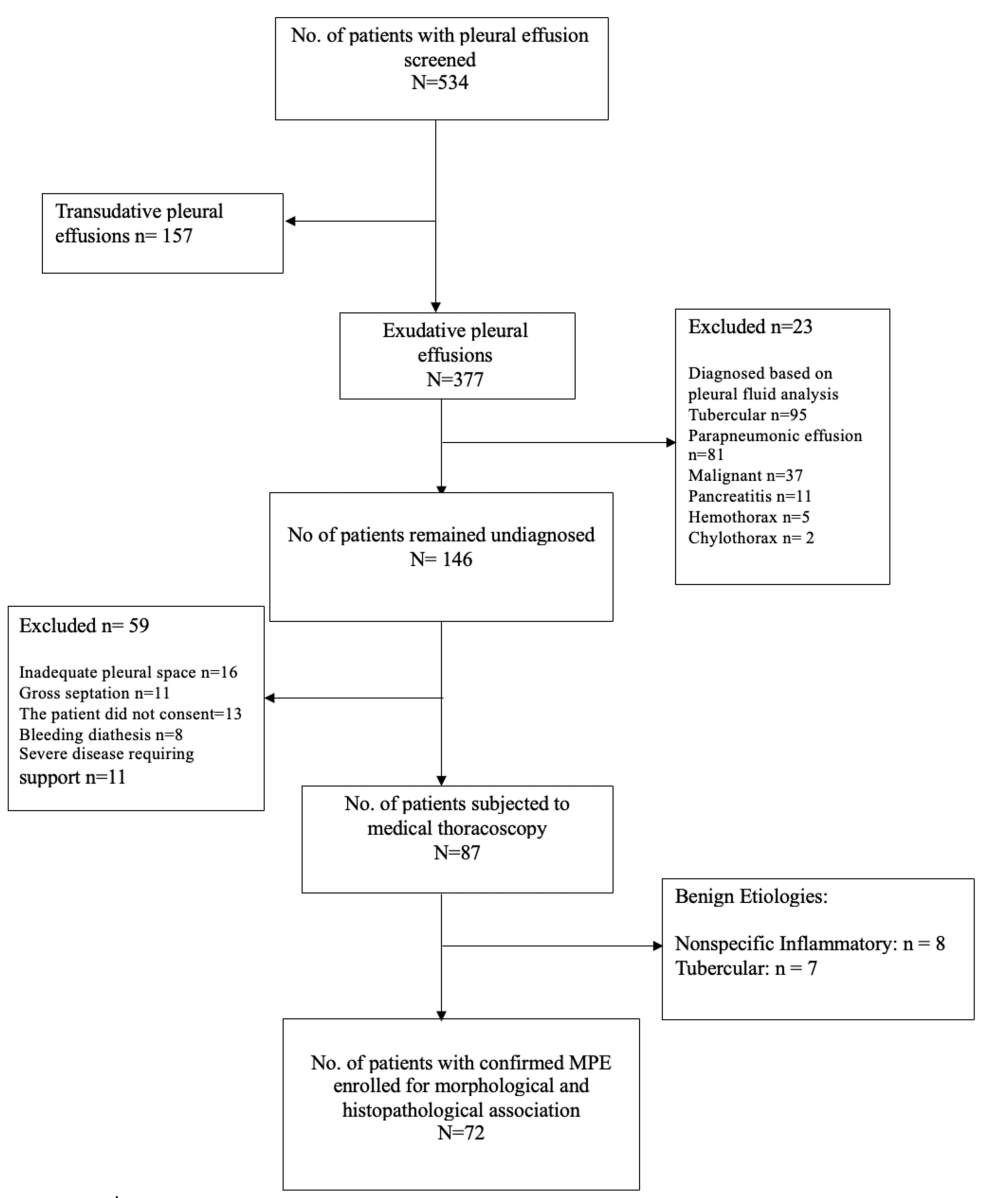
Flowchart of patient selection MPE: malignant pleural effusion

All patients with pleural effusion were evaluated and screened at the Department of Pulmonary Medicine, VIMSAR, India. Following thorough examinations and pleural fluid analysis, the feasibility of the thoracoscopic procedure was assessed for those who remained undiagnosed. Once found feasible, patients have been informed of the need for and complications of thoracoscopy. Screened undiagnosed pleural effusions were subjected to pleural biopsy using a rigid thoracoscope. Pregnancy, patients with bleeding diathesis, platelet count <50,000/cc, deranged liver function, deranged renal function, inadequate pleural space, and gross septation on ultrasonography were excluded from the study. The patients diagnosed with malignancy after MT were analyzed for morphological and histopathological association. A total of 87 patients were recruited, among whom 72 patients had confirmed pleural malignancies. The detailed patient selection flowchart is shown in Figure 1.

Study procedure

**Figure 2:**
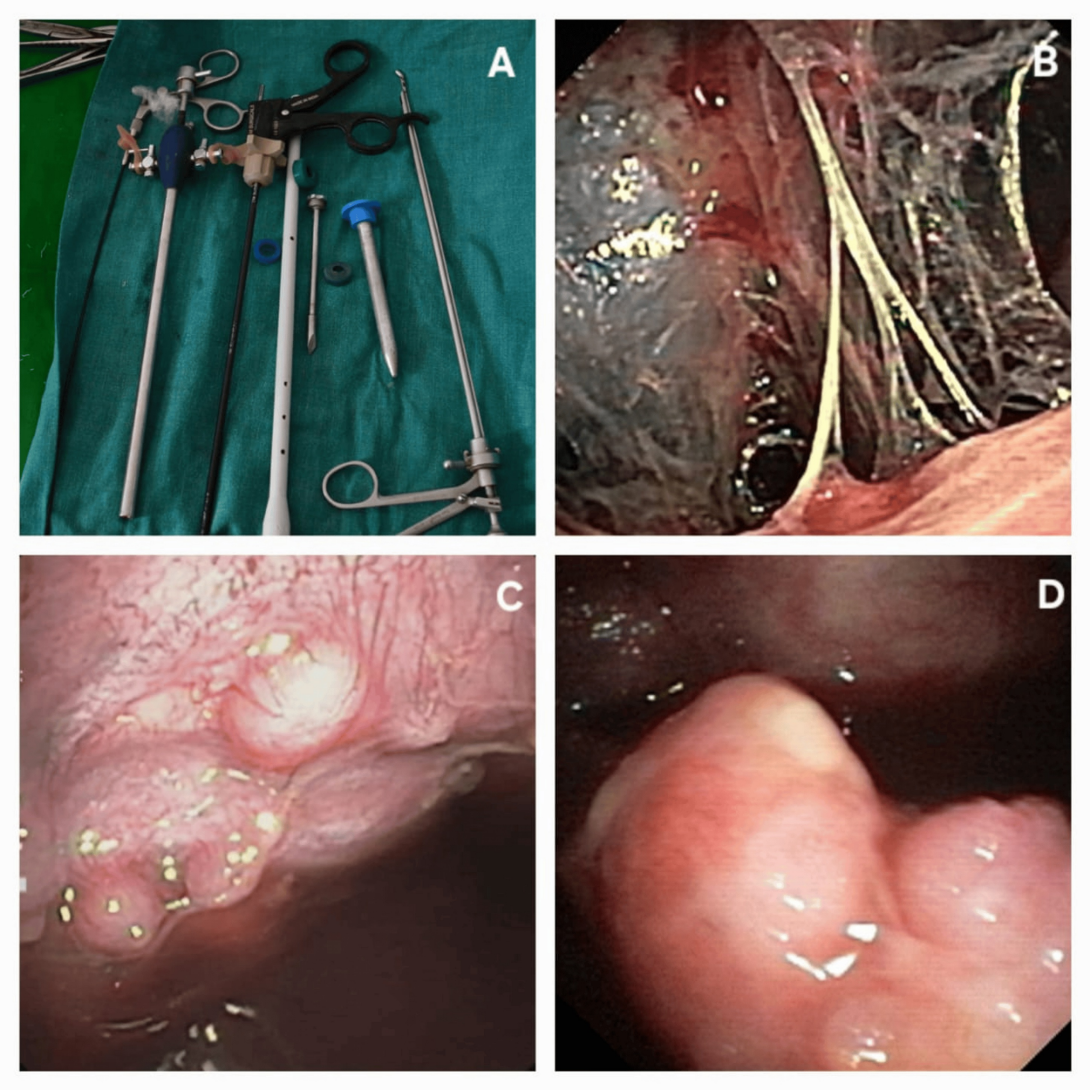
Medical thoracoscope and various morphological patterns in pleural space A: rigid thoracoscope and accessories, B: strands, C: nodules, D: mass

Written informed consent was taken before each procedure. A detailed history and physical examination was done. The history of any comorbidities was recorded in the pre-structured data sheet. Medical thoracoscopy was performed within the procedure suite using a rigid thoracoscope (Optymed®, Delhi, India) of 7 mm outer diameter, 300 mm length, and oblique optics, with a stainless steel valved trocar (Figure 2A). The procedure was performed in lateral decubitus position, keeping the side of intervention upward while the patient faced the operator. The overlying skin was scrubbed with povidone-iodine and propyl alcohol solutions and draped with utmost asepsis. We performed MT under local anesthesia. Two percent lidocaine with adrenaline was used generously to anesthetize skin, subcutaneous fascia, muscle plane, periosteum, and parietal pleura in an ordered fashion, keeping the dose below 4 mg/kg. The usual dose was around 20 ml of 2% lidocaine. Midazolam (0.1 mg/kg) and nalbuphine (0.2-0.3 mg/kg) were used for sedation and analgesia during the procedure. A skin incision of size 1.5-3 cm was usually given on the skin to expose the subcutaneous fascia. The path to the pleural space was dissected bluntly to reach the pleural space. If required, the incision was extended, and the path for the trocar was felt with the little finger. Further, the trocar was placed gently in a corkscrew motion. The rigid scope was introduced gently to inspect the pleural space. Excess pleural fluid used to be drained in a graded manner using negative suction. Intermittently, free air was allowed to enter the pleural space to avoid rapid expansion of the lung and maintain adequate pleural space for intervention. The pleural cavity was inspected systematically, like the costal, diaphragmatic, mediastinal, and apical pleura along with gutters. Any anatomical breach and pathology found were noted down. If found, lesions like masses, plaques, nodules, fibrinous strands, and adhesions were noted down (Figure 2B-2D). The nodules were tiny, distinct eruptions from pleura that can be plucked completely from the base with minimal effort. Mass lesions were defined as large and irregular outgrowths, often have a broad base and can't be completely plucked using the standard thoracoscopic biopsy forceps. Plaques are thickened, flat fibrous patches. Adhesions were defined as abnormal parietal and visceral pleural adhered together. Strands were defined as fibrinous bands or sheets that stretch between pleura to other near by structures. Routinely, we took four to six chunks of biopsy from the lesions in the “pinch and peel” technique. The samples were collected in formalin and normal saline containing containers for histopathologic evaluation, bacteriological and mycobacterial evaluations. 

We put a chest drain post-biopsy through the tract made earlier. Any immediate complications were noted down. Vitals like heart rate, continuous electrocardiogram (ECG), oxygen saturation (SpO2), and blood pressure were monitored throughout the procedures, and necessary management was provided if noticed. Biopsy samples were sent for pathological evaluation in formalin and saline for bacteriological assessment. Paracetamol and diclofenac injections were used for post-procedural pain management as per requirement. Patients were kept in the ward for at least 24 hours to observe early procedural complications. We followed patients for 28 days over the telephone to check for any complications. All the information was filled in on a pre-structured data sheet for future reference.

Statistics

All the data were entered in SPSS for Windows version 20.0 (IBM Corp., Armonk, NY, USA) and analyzed. The categorical data were mentioned in numbers and percentages. The continuous data were subjected to a normality check using Kolmogorov-Smirnov tests. The normally distributed data were presented with mean and standard deviations. Non-parametric data were presented as the median and interquartile range (IQR). The association between different categorical variables was checked by the chi-squared test or Fisher’s exact test. p-value <0.05 was taken as statistically significant in the study.

## Results

Following the initial pleural fluid examination, 146 (27.3%) of the 534 individuals we assessed for pleural effusion did not receive a diagnosis. MT was carried out on 87 people. Fifteen patients were diagnosed with a benign etiology. Morphological and histological associations were analyzed in 72 patients diagnosed with MPE.

Patient characteristics

The mean age of the population was 54.17 years. Elderly, male, ever smokers were the predominant subjects of the cohort. We observed majority of patients presented with shortness of breath, chest pain, and cough. Most patients presented late with a mean duration of more than one month. The detailed characteristics of the study population are provided in Table 1.

**Table 1:** Baseline characteristics of the study population SD: standard deviation, ADA: adenosine deaminase, LDH: lactate dehydrogenase, USG: ultrasonography, ICD: intercostal drainage, IQR: interquartile range, VAS: visual analogue scale, MT: medical thoracoscopy, SBP: systolic blood pressure, SpO2: saturation of oxygen

Baseline Demography	N=87
Age in years (mean ± SD)	54.17 (±⁣15.17)
Gender	
Male(n,%)	46 (52.88%)
Comorbidities	
Hypertensive(n,%)	16(18.39%)
Diabetics(n,%)	9(10.34%)
Smoking status	
Non-smokers(n,%)	37(42.52%)
Ever smokers(n,%)	50(57.47%)
Symptoms	
Duration of symptoms in days (Mean±SD)	43.47(±10.80)
Cough(n,%)	51(58.62%)
Chest pain(n,%)	54(62.06%)
Shortness of breath(n,%)	56(64.36%)
Loss of appetite(n,%)	9(10.34%)
Weight loss more than 5 kgs(n,%)	20(22.98%)
Fever(n,%)	24(27.58%)
Hemoptysis(n,%)	2(2.29%)
Clubbing (n,%)	39(44.82%)
Pallor(n,%)	32(36.78%)
Duration of symptoms before presentation	
1-30 days(n,%)	28(32.18%)
>30 days(n,%)	59(67.82%)
Baseline pleural effusion characteristics	
Side of the effusion	
Right Pleural effusion(n,%)	38(43.68%)
Left pleural effusion(n,%)	39(44.83%)
Bilateral pleural effusion(n,%)	10(11.49%)
Colour of pleural fluid	
Straw (n,%)	19(21.83%)
Haemorrhagic (n,%)	63(72.41%)
Turbid (n,%)	5(5.74%)
Biochemical tests	
Hemoglobin in g/dL (Mean±SD)	10.27(±2.16)
Pleural fluid Glucose in mg/dL (Mean±SD)	78.72(±24.74)
Pleural fluid protein in g/dL (Mean±SD)	4.77(±1.21)
Pleural fluid ADA in U/L (Mean±SD)	34.59(±16.43)
Pleural fluid LDH in mg/dL (Mean±SD)	1439.78(±464.00)
Pleural fluid amylase in U/L (Mean±SD)	37.91(±13.94)
Procedure related parameters	
Maximum vertical depth measured by USG in cm (Mean±SD)	8.97(±2.12)
Amount of effusion drained in ml (Mean±SD)	1580(±102.2)
Duration of procedure in minutes (Mean±SD)	42.22(±12.69)
Change in Systolic BP in mmHg (Mean±SD)	6.67(±9.19)
Maximum dip in saturation of oxygen (Mean±SD)	5.13(±3.76)
Time to removal of ICD (Median with IQR)	8(6-11)
Pain VAS score(Median with IQR)	5(4-6)
Histopathological diagnosis	
Tuberculous pleural effusion	7 ( 8.5%)
Malignant Pleural effusion	72 (82.7%)
Nonspecific pleural effusion	8 (9.2%)
Complications related to MT	
Pain at the incision site(n,%)	87 (100%)
Fall in SBP ≥ 10 mm Hg(n,%)	21(24.17%)
Mean fall in SBP	6.6±9.25
Fall in Spo2 ≥ 5%, (n,%)	34(39.08%)
Mean fall in SpO_2,_ (Mean±SD)	5.13±3.78
Minor bleeding (n,%)	17(19.65%)
Major bleeding (n,%)	0(0.0%)
Subcutaneous emphysema(n,%)	0(0.00%)
Post thoracoscopic fever(n,%)	24(31.03%)
Prolonged air leak(n,%)	0(0.00%)
Infection(n,%)	1(1.14%)
Residual pneumothorax(n,%)	0(0.0%)
28 day mortality(n,%)	0(0.00%)
Macroscopic appearances in MT	N=72
Nodules(n,%)	46(63.9%)
Masses(n,%)	26(36.1%)
Plaques(n,%)	22(30.6%)
Strands (n,%)	19(26.4%)
Adhesion(n,%)	27(37.5%)

Pleural fluid biochemical reports

Thirty-nine (44.83%) patients had left-sided effusion, and 38 (43.68%) had right-sided effusion. Sixty-three (72.41%) cases had hemorrhagic pleural effusions. The mean glucose level in pleural fluid was 78.72 (SD: +/-24.74) gm/dl. Eighty-two (94.2%) patients had a pleural fluid lactate dehydrogenase (LDH) level of more than 1000 u/L, and in 66 (75.86%) cases, the fluid protein level was more than 4 g/dL. Most of the cases (57, 65.55%) had pleural fluid adenosine deaminase (ADA) levels < 40 U/L. On pleural fluid cytological analysis among MPE (n=72), 70 (97.2%) were lymphocyte predominant. Cytology was positive for suspicious malignant cells in 22 (30.5%) cases among MPE. The detailed characteristics are mentioned in Table 1.

Diagnostic yield of medical thoracoscopy

Out of 87 patients who underwent the procedure, 90.8% (95% CI: 84.8%-96.8%) of patients reached a definitive diagnosis. Seventy-two patients had malignant effusions, and seven patients had tuberculous pleural effusions. The remaining eight patients remained undiagnosed, considering nonspecific inflammation.

Complications associated with rigid medical thoracoscopy

The most common complication was pain at the incision site, followed by a dip in room air saturation of oxygen. More than a 5% drop in oxygen saturation was noted in 34 (39.1%) of cases. A fall in systolic blood pressure of more than 10 mm Hg was noted in 21 (24.13%) of cases. Only two (2.29%) patients required vasopressor support after the procedure, which subsequently improved. Pain associated with the procedure was mild (5, IQR: 4-6). In our study, no patient died within four weeks of the procedure. Other relevant complications are mentioned in Table 1.

Thoracoscopic findings and histopathological association in MPE

In our study, metastatic adenocarcinoma (45, 62.5%) was the most common histopathological finding. Multiple morphological findings were appreciated in the pleural space during MT (Table 2).

**Table 2:** Spectrum of etiologies obtained for MPE MPE: malignant pleural effusion

Histopathology	Counts	Percentages
Metastatic adenocarcinoma	45	62.5%
Lymphoma	9	12.5%
Malignant mesothelioma	8	11.1%
Small cell carcinoma	5	6.9%
Undifferentiated carcinoma	4	5.6%
Squamous cell carcinoma	1	1.4%

We observed pleural nodules (46, 63.9%) followed by mass lesions (26, 36.1%). Other findings were that plaques, adhesions, and fibrinous strands were seen in 22 (30.6%), 27 (37.5%), and 19 (26.4%) of cases, respectively.

Metastatic adenocarcinoma was associated with a higher percentage of nodules (39, 86.67%; p<0.01). However, we did not observe nodules as a predominant morphological character in lymphoma (5, 62.50%; p=0.57), malignant mesothelioma (2, 25.00%; p< 0.01), small-cell carcinoma (0, 0.0%; p<0.01), and undifferentiated carcinoma (0, 0.0%, p<0.05). The histopathological and morphological association is depicted in Table 3. The significant morphological findings of malignant mesothelioma were plaques (8, 100%, p<0.01) and adhesions (8, 100%, p<0.01). In our study, lymphomas (8, 88.89%; p<0.01), and undifferentiated carcinomas (4, 100%; p<0.01) were associated with mass lesions. In small-cell carcinoma, we found a significant association with strands (5, 100%; p< 0.01), but metastatic adenocarcinoma had a negative association with strands (6, 13.34%; p<0.01).

**Table 3:** Distribution and association of different types of lesions seen in MT with different types of malignancies MT: medical thoracoscopy *: p<0.05: considered significant in this study.

Morphologies across different histopathologies	Number (n)	Percentage (%)	p-value	Chi-square
Metastatic adenocarcinoma	45	100		
Nodules	39	86.67	0.01*	26.98
Mass	6	13.33	0.01*	26.98
Plaques	14	31.11	0.89	0.01
Adhesions	16	35.56	0.66	0.19
Strands	6	13.33	0.01*	10.53
Lymphoma	9	100		
Nodules	5	62.50	0.57	0.31
Mass	8	88.89	0.01*	12.41
Plaques	0	0.00	0.04*	4.52
Adhesions	3	33.33	0.78	0.08
Strands	3	33.33	0.62	0.25
Malignant Mesothelioma	8	100		
Nodules	2	25.00	0.01*	5.90
Mass	4	50.00	0.39	0.75
Plaques	8	100.00	0.01*	20.45
Adhesions	8	100.00	0.01*	15.00
Strands	2	25.00	0.92	0.01
Small cell carcinoma	5	100		
Nodules	0	0.00	0.01*	9.51
Mass	3	60.00	0.24	1.33
Plaques	0	0.00	0.12	2.36
Adhesions	0	0.00	0.07	3.23
Strands	5	83.34	0.01*	14.98
Undifferentiated carcinoma	4	100		
Nodules	0	0.00	0.01*	7.49
Mass	4	100.00	0.01*	7.49
Plaques	0	0.00	0.17	1.86
Adhesions	0	0.00	0.11	2.54
Strands	3	75.00	0.02*	5.15
Squamous cell carcinoma	1	100		
Nodules	0	0.00	0.18	1.79
Mass	1	100.00	0.18	1.79
Plaques	0	0.00	0.50	0.44
Adhesions	0	0.00	0.43	0.60
Strands	0	0.00	0.54	0.36

## Discussion

Our study highlighted the association between types of lesions in the pleural space and the associated histopathology of the malignant pleural lesions. This study further established that MT is relatively safe. Considering histopathology as the gold standard, thoracoscopy provides a good chunk of tissue for cancer evaluation. Rigid medical thoracoscopy helped to establish the diagnosis in 80 (54.42%) cases with undiagnosed effusions (n=146). We found that 72 (82.76%) cases subjected to thoracoscopy were malignant. In this study, the diagnostic yield of rigid MT was at least 90.80% (95% CI: 84.8%-96.8%). Due to a lack of precise diagnostic values, patients with nonspecific inflammation in histopathology were not considered for diagnosis. The individuals with nonspecific inflammation were not monitored for long enough for us to determine the exact diagnosis of the conditions. If it had been adhered to, the diagnostic yield was anticipated to rise. Various authors have demonstrated similar results in the literature [9,10], which suggests medical thoracoscopy has an undisputed diagnostic value in a pleural sampling method.

We observed that the most common procedure-related complication was pain at the incision site. Other complications were minor desaturation, a fall in systolic blood pressure, and minor bleeding. Only one patient developed empyema, which was managed with antibiotics and subsequently improved. Hansen et al. observed similar results [11]. Patil et al. observed prolonged air leaks for more than seven days in 4.6% of cases, followed by subcutaneous emphysema by a two-port rigid thoracoscopy [6]. Nattusamy et al. reported localized subcutaneous emphysema in 16.67% and mild bleeding in 4.17% of cases using a semi-rigid thoracoscopy [7]. We did not notice any major bleeding requiring blood transfusion, cardiac arrhythmia, or procedure-related mortality.

Thirty-seven cases were non-smokers, out of which 33 (89.19%) were females, suggesting that apart from smoking, other risk factors also play a significant role in cancer biogenesis. Pleural involvement in malignancy demonstrates advanced-stage disease. Pleural fluid drainage improves the patient’s quality of life by improving dyspnea. Along with tissue sampling, MT helps to alleviate dyspnea by draining the pleural space and placing intercostal tubes under a panoramic vision. Understanding the morphological characterization of malignant lesions may help in going for advanced procedures like pleurodesis in the same setting. Various types of macroscopic features (nodules, masses, plaques, and adhesions) can be appreciated while inspecting the pleura during MT. Different malignancies are associated with different lesion types. Also, considerable overlapping exists among morphological patterns and histopathologies. This is similar to the findings of other studies that demonstrated that thoracoscopic findings of MPE mostly showed nodules [8-10,12].

The majority of biopsy specimens had adenocarcinoma on histopathology in this study. Different authors demonstrated that the most common cancer metastasizing to the pleura was adenocarcinoma, ranging from 21 to 67% [6-8]. The specific organ of origin of adenocarcinoma has not been evaluated in this study due to logistical constraints.

Metastatic adenocarcinoma was associated with a higher percentage of nodules, followed by adhesion and plaques. This is quite similar to the study done by various authors [8,13]. The implantation theory of pleural metastasis and the hematogenous spread of the disease may be the cause for the higher number of nodules in metastatic adenocarcinoma [14,15]. Lymphoma presented with a higher percentage of masses (88.9%). Similar to our observations, Abumossalam et al. mentioned a higher percentage of nodules and masses in lymphoma undergoing MT [8]. The higher number of masses and nodules in lymphoma is probably because of dense pleural lymphatic circulation and the local spread of the disease. 

We observed plaques and pleural thickening in 100% of cases with malignant mesothelioma. Malignant mesothelioma is a neoplastic lesion of the pleura, and a higher number of plaque-like lesions were expected. For small cell carcinoma, we noticed strands and plaques as the only findings in our study, which are quite different from another study [8]. Undifferentiated carcinoma presented predominantly with mass-like lesions. In our study, we had only one case of metastatic squamous cell carcinoma, which presented as a mass lesion. We noted multiple types of lesions in metastatic adenocarcinoma and malignant mesothelioma. Having more than one lesion in the pleura helps to take adequate pieces for histopathology and improve the yield.

We attempted to segregate the morphological appearance observed while performing MT. The study will also give an idea for future research on the standardized morphological description of thoracoscopic findings. Additionally, it is important to investigate whether lesion characteristics, lesion counts, and the overlap of various lesion patterns have any bearing on survival. The study has several limitations. It was a cross-sectional, single-center study with a relatively small sample size in several subgroups. As morphological descriptions are not standardised and difficult to describe, there is a fair chance for interobserver variability. We did not emphasize searching for the primary sites of the disease, as we referred the patients to the oncology team for further evaluation once the tissue diagnosis was reached. Due to a lack of an immunohistochemistry facility, it could not be done; hence, we were not able to subtype the malignancies. Long-term follow-up could not be arranged to understand the long-term outcome of the patients who underwent MT. The major strength was the prospective nature of the study.

## Conclusions

Medical thoracoscopy remains a pivotal diagnostic modality in the evaluation of malignant pleural effusion. It is minimally invasive and, combined with high diagnostic yield, enables direct visualization and targeted pleural biopsies, thereby facilitating early and accurate diagnosis. Histopathological and morphological analyses of the thoracoscopic biopsy specimens not only confirm the malignancy but also provide crucial insights into tumor type and extent of pleural involvement. The association between thoracoscopic findings and histopathological patterns may enhance diagnostic precision and further oncological management. Continued refinement of morphological description, along with multicentric validation, may work like a pathological marker. Subsequently, incorporating it into clinical pathways to increase the diagnostic accuracy may enhance patient outcomes in malignant pleural effusions. 
